# Metabolites of Astragalus membranaceus and their pro-apoptotic and cytotoxic activities: insights into targeted metabolic pathways

**DOI:** 10.3389/fphar.2025.1647958

**Published:** 2025-08-29

**Authors:** Jie Liu, Dongwei Wang, Na Ren, Li Zhang, Ting Wang

**Affiliations:** ^1^ Office of Shandong College of Traditional Chinese Medicine, Yantai, China; ^2^ Department of Proctology, Jinan Huaiyin People’s Hospital, Jinan, China; ^3^ Pharmacy Department, Jinan Municipal People’s Government Organs Outpatient Department, Jinan, China; ^4^ Pharmacy Department, Community Health Service Centre of Guyunhu Street, Jinan, China; ^5^ Department of Pharmacy, Jinan Second People’s Hospital, Jinan, China

**Keywords:** Astragalus membranaceus, active metabolites, metabolic pathways, mechanism, therapeutic targets

## Abstract

Astragalus membranaceus (Astragalus), a traditional Chinese herbal medicine, is well known for its immunomodulatory effects. Recent studies have demonstrated that Astragalus exhibits antiviral, anti-inflammatory, anti-aging, anti-atherosclerotic, antioxidant, and immune-enhancing activities, as well as pro-apoptotic and cytotoxic effects on tumor cells. It is increasingly used as an adjuvant therapy in oncology. The mechanisms underlying its pro-apoptotic and cytotoxic activities include inhibition of tumor cell proliferation and migration, modulation of tumor-associated metabolic pathways, induction of tumor cell apoptosis, cell cycle arrest, regulation of autophagy, targeting of the tumor microenvironment, inhibition of neo-angiogenesis, and enhancement of host immunity. This review provides a comprehensive summary of the active metabolites of Astragalus and their pro-apoptotic and cytotoxic mechanisms, with a focus on metabolic regulation, offering a theoretical basis for its rational application in tumor therapy. Future research aimed at precise metabolite-guided interventions could improve patient outcomes and quality of life.

## 1 Introduction

Cancer is a major contributor to the global burden of disease, and global cancer incidence and mortality will continue to increase significantly over the next 30 years. As a highly fatal malignant disease, cancer poses tremendous treatment challenges and mortality rates, imposing heavy burdens on patients and families (Dee et al., 2025; Wu et al., 2024). Unfortunately, cancer accounts for about one-sixth of global deaths, and its mortality rate remains high (Bizuayehu et al., 2024). According to the latest epidemiological reports, by 2020, there will be nearly 20 million new cancer cases worldwide, and nearly 10 million deaths from cancer recurrence and metastasis (He S. et al., 2024a). Epidemiologic studies in China have found that globally, China accounts for about 24% of the world’s new cancer cases and about 30% of the world’s total cancer deaths (Qi et al., 2023). Current mainstream cancer treatments remain surgery, chemotherapeutic drugs (alkylating agents, antimetabolites, antitumor antibiotics, antitumor hormones, or physical radiotherapy (α, β, γ rays, and various X-rays) (Wolf et al., 2024; Feng et al., 2025; Odeny et al., 2024). Although these treatments demonstrate certain efficacy, they also induce numerous adverse effects (immune function suppression, bone marrow suppression, hematopoietic dysfunction, alopecia, skin/mucosal damage, *etc.*) (Zafar et al., 2024; Huang et al., 2023). These severe side effects significantly compromise clinical therapeutic outcomes and diminish cancer patients’ quality of life. Traditional Chinese Medicine (TCM) offers a holistic approach to cancer treatment, guided by the principles of “supporting healthy qi (vital energy)” and “eliminating pathogenic factors.” TCM aims to modulate immune function and promote cancer cell apoptosis, thereby compensating for the limitations of Western cancer therapies (Sajeev et al., 2024; Li S. et al., 2025a). Modern pharmacological research has demonstrated that Chinese medicinal herbs, such as Astragalus, Hedyotis diffusa, and Scutellaria barbata, possess antitumor properties. These herbs exhibit capabilities including immune enhancement, improvement of patient constitution, and mitigation of adverse reactions induced by chemotherapy (Dong et al., 2023; Huang et al., 2022; Li Y. et al., 2022a).

Astragalus is a quintessential medicine–food homology (MFH) plant. Its root is commonly used in Chinese soups and tonics, and it is rich in polysaccharides, flavonoids, and saponins. These compounds ensure both nutritional safety and potent immunomodulatory, anti-inflammatory, and antitumor activities. This duality makes Astragalus highly valuable in studies focusing on tumor immune microenvironment regulation and adjunctive cancer therapy (Meng-Yao Li et al., 2025). Wild Astragalus roots are harvested in autumn, while cultivated Astragalus is collected 4–5 years after planting, either before spring germination or after autumn defoliation, followed by removal of stems and fibrous roots before drying (Sheng et al., 2024). Astragalus grows in hilly shrublands and sandy loam areas of dry slopes, primarily distributed in China’s Heilongjiang, Jilin, Liaoning, and Hebei provinces. Astragalus thrives in mountainous wilderness, mainly found in Heilongjiang, Jilin, Inner Mongolia, Hebei, Shanxi, and Tibet. The medicinal use of Astragalus was first documented in Shennong’s Classic of Materia Medica and has been inherited through generations of TCM practitioners (He Z. et al., 2024b). The Compendium of Materia Medica records: Sweet in taste, slightly warm in nature; thin in qi but rich in flavor; ascending and descending properties; yang within yang; non-toxic. Specialized in qi supplementation. Enters the lung, spleen, and heart meridians. While having multiple therapeutic functions, its unique efficacy particularly manifests in blood nourishment (Li C. X. et al., 2022b). Active metabolites of Astragalus include saponins, polysaccharides, flavonoids, folic acid, and trace elements such as selenium, zinc, and copper (Li S. et al., 2020a). These natural bioactive compounds from Astragalus provide novel perspectives for antitumor research. Extensive clinical and mechanistic studies have demonstrated that Astragalus possesses immunomodulatory (Wang et al., 2020) and tumor growth inhibitory effects (Yang et al., 2024), and is now widely used as adjuvant therapy for various malignant tumors.

This review comprehensively summarizes the relevant research on the main metabolites and safety characteristics of Astragalus in antitumor activity over the past decade, as well as the relevant research on its active metabolites, action pathways, and antitumor mechanisms. These theoretical foundations are expected to provide a solid basis for future research on the antitumor mechanisms and clinical applications of Astragalus, while also offering new insights for the development of precision medicine.

A systematic literature search was conducted to comprehensively collect relevant studies on the metabolites and anti-tumor applications of *Astragalus membranaceus*. Multiple electronic databases, including PubMed, Web of Science, Scopus, and CNKI, were searched from inception to the date, using a combination of keywords such as “Astragalus membranaceus,” “anti-tumor,” “pro-apoptotic and cytotoxic effects,” “saponins,” “polysaccharides,” “flavonoids,” and “metabolic pathways.” Both English and Chinese language articles were considered to ensure a broad coverage.

Inclusion criteria were: (1) original research articles, reviews, or clinical trials focusing on the chemical metabolites and anti-tumor mechanisms or clinical applications of *Astragalus membranaceus*; (2) studies involving *in vitro*, *in vivo*, or human clinical data; (3) articles published in peer-reviewed journals. Exclusion criteria included conference abstracts, non-peer-reviewed publications, and studies lacking sufficient experimental detail or relevance.

After initial screening based on titles and abstracts, full texts of potentially eligible articles were retrieved and assessed independently by two reviewers. Discrepancies were resolved through discussion or consultation with a third reviewer. Data extraction focused on study design, experimental models, dosage and preparation of Astragalus, outcomes related to anti-tumor efficacy, and mechanistic insights.

This methodical approach ensured a rigorous and reproducible literature selection process, thereby providing a reliable foundation for the comprehensive review presented in this manuscript.

## 2 Anti-tumor active bioactive metabolite of Astragalus

Pharmacological studies have found that crude extracts and isolated metabolites of Astragalus can be effectively used for anti-inflammatory, immune stimulation, antioxidant, pro-apoptotic and cytotoxic effects, antidiabetic, cardioprotective, hepatoprotective, and antiviral activities (Table 1). Modern research has demonstrated that Astragalus possesses antiviral, anti-inflammatory, anti-aging, anti-atherosclerotic, antitumor, antioxidant, and immune-enhancing effects (Auyeung et al., 2016; Liu et al., 2017). Saponins, flavonoids, and Astragalus polysaccharides are the main active metabolites responsible for its antitumor effects (Tian et al., 2025; Tang and Huang, 2022). With the rapid development of technology, the purification techniques for Astragalus have also advanced significantly in recent years. Over 200 compounds have been isolated and identified from Astragalus, primarily classified into polysaccharides, flavonoids, saponins, and amino acids, along with small amounts of alkaloids, lignans, sterols, and various trace elements (Liu et al., 2024). The stems, leaves, and flowers of Astragalus contain similar levels of active metabolites as its roots, including polysaccharides, saponins, flavonoids, and amino acids, showing promising prospects for research and development (Wang et al., 2019; Hao et al., 2023).

**TABLE 1:** Major active metabolites of Astragalus and their pharmacological effects.

Active metabolites	Representative compounds	Pharmacological activities
Saponins	Astragaloside I–IV, Isoastragaloside IV, Cycloastragenol	Antitumor, Immunomodulatory, Anti-EMT, Chemosensitizing
Polysaccharides (APS)	Astragalus polysaccharides	Immunostimulatory, Antioxidant, Anti-aging, Tumor apoptosis, Reverses drug resistance
Flavonoids	Formononetin, Calycosin-7-O-β-D-glucopyranoside	Anti-inflammatory, Antitumor, Cardioprotective, Anti-atherosclerotic
Others	Amino acids, Alkaloids, Sterols, Trace elements	Antiviral, Nutritional support, Hepatoprotective

### 2.1 Saponins

Saponins often exist in free forms and exhibit relative pharmacological effects and strong biological activity. Due to the difficulty in breakthroughs in purification and isolation techniques for Astragalus saponins, related research progress has been slow. Saponin compounds possess biological activities such as antiviral, antitumor, and immunomodulatory effects (Zhang et al., 2022). The main saponin metabolites of Astragalus include astragalosides I–VIII, isoastragalosides I, II, and IV, cycloastragenols E, F, and G, and astragaloside I–IV, among others (Luo et al., 2022; Nafti et al., 2022). Among these, astragaloside IV is a marker metabolites for quality control of Astragalus and one of the primary substances responsible for its antiviral pharmacological activities. Studies have found that it can induce tumor cell apoptosis, suppress tumor cell migration and invasion, inhibit epithelial-mesenchymal transition (EMT), regulate autophagy, modulate immune responses, reduce toxic side effects during tumor treatment, and enhance sensitivity to antitumor drugs (Yao et al., 2023; Ziyang et al., 2023).

The doses of astragaloside IV used in *in vitro* studies typically range from 1 to 50 μM, with treatment durations varying between 24 and 72 h. Both human cancer cell lines (e.g., MCF-7, A549) and animal models have been utilized to investigate its effects. Positive controls often include standard chemotherapeutic agents, while untreated cells or vehicle-treated groups serve as negative controls. These well-controlled designs ensure the observed effects are attributable to the saponins (Wang J. et al., 2021b; Zhao et al., 2020).

### 2.2 Polysaccharides

Astragalus polysaccharide (APS), a natural active metabolites extracted from Astragalus, is a macromolecular substance that plays a decisive role in its pharmacological effects. APS exhibits biological activities such as antiviral, immunomodulatory, antioxidant, anti-aging, anti-inflammatory, antitumor, and anti-atherosclerotic properties (Zhang Q. et al., 2024b; Li W. et al., 2020b; Zhong et al., 2022). Extensive research has proven that APS not only promotes tumor cell apoptosis but also enhances immunity to exert antitumor effects. Additionally, APS can improve the efficacy of chemotherapy drugs and reduce tumor cell drug resistance, making its antitumor role increasingly prominent in the field of oncology (Cao et al., 2024).

Typical APS doses *in vitro* range from 50 to 400 μg/mL, applied over 24–72 h, depending on the cell type and endpoint. *In vivo* models include murine tumor-bearing mice treated with APS doses from 50 to 200 mg/kg via intraperitoneal or oral administration for periods of 1–4 weeks. Controls include untreated and placebo groups, while some studies employ standard immunomodulators or chemotherapy drugs as positive controls. These parameters clarify the experimental contexts in which APS exerts its effects (Li et al., 2019; Chen et al., 2022a).

### 2.3 Flavonoids

**FIGURE 1:**
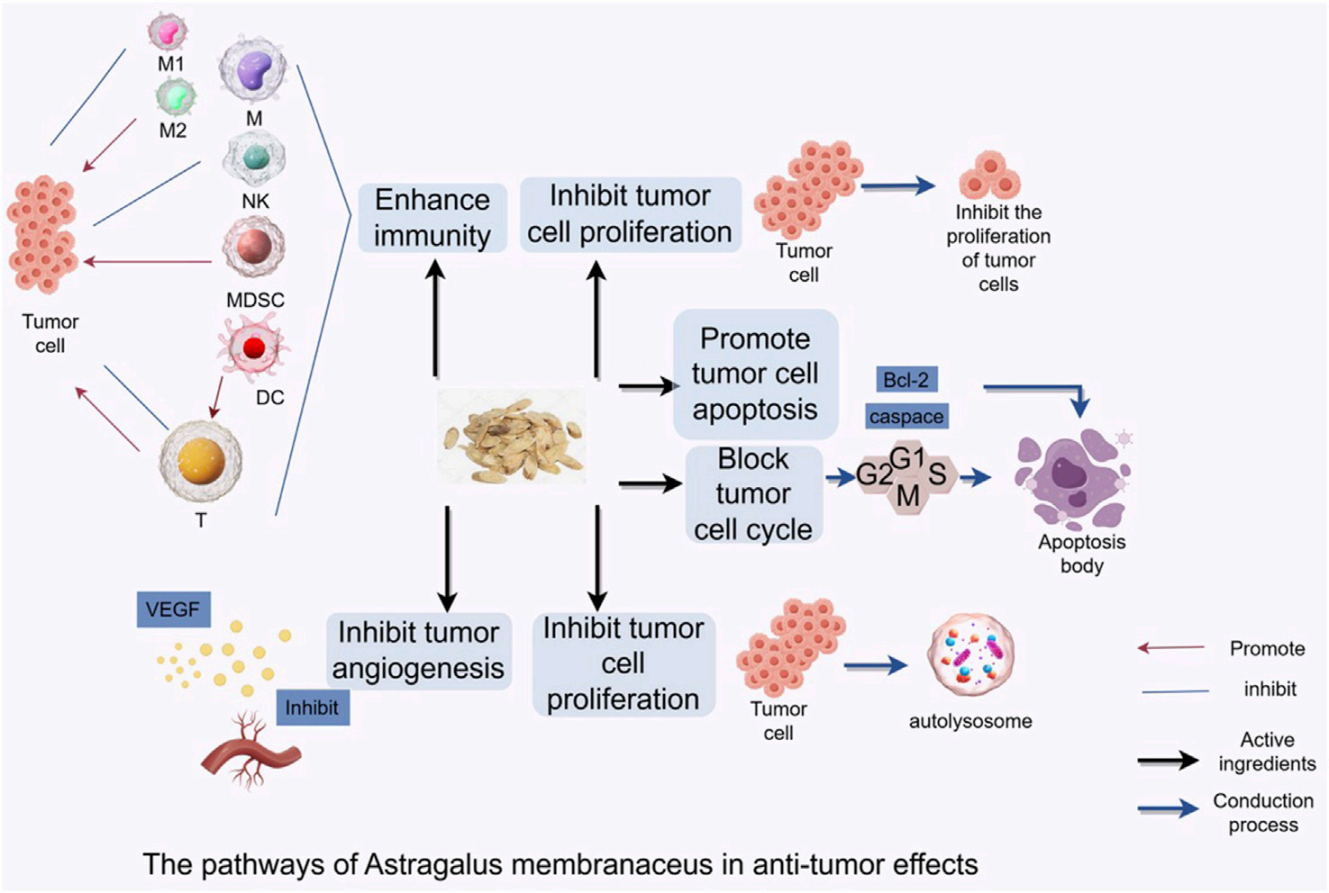
Astragalus Mongholicus inhibits tumorigenesis and progression by regulating signaling pathways through regulating cell cycle, inducing tumor cell autophagy, modulating tumor microenvironment, and enhancing anti-tumor immune function.

Flavonoids are one of the key active metabolites of Astragalus, with pharmacological effects including anti-inflammatory, antitumor, hypoglycemic, immunomodulatory, and antiviral activities (Yang et al., 2023). Over 50 flavonoid compounds have been purified from Astragalus, including flavonoid aglycones, isoflavones and their glycosides, isoflavans, and pterocarpans, with isoflavones and their glycosides being the most abundant (Sheng et al., 2021; Cui et al., 2022). Important active flavonoid metabolites in Astragalus include formononetin and calycosin-7-O-β-D-glucopyranoside (Xu et al., 2022; Li M. et al., 2022c; Liu F. et al., 2020a). The Astragalus isoflavone formononetin protects cardiomyocytes through antioxidant, anti-apoptotic, and autophagy-promoting effects, demonstrating excellent results in cardiovascular system protection (Chen Q. et al., 2024a). Furthermore, related studies have shown its beneficial protective effects in anti-atherosclerosis. Astragalus and its different metabolites inhibit tumorigenesis and progression by modulating multiple signaling pathways (Figure 1).

*In vitro* experiments with formononetin typically utilize concentrations ranging from 5 to 40 μM, with treatment durations of 24–72 h on various tumor and normal cell lines. *In vivo* studies administer doses of 10–50 mg/kg via oral or intravenous routes, over time frames from several days to weeks. Controls include vehicle treatment and known pathway inhibitors. Such detailed experimental setups validate the flavonoids’ multifaceted biological effects (Zhang et al., 2021; Liu J. et al., 2020b).

## 3 Anti-tumor mechanisms of Astragalus

### 3.1 Inhibition of tumor cell proliferation and migration

Tumor cells are characterized by uncontrolled proliferation, infinite growth, and invasive metastasis. Reducing tumor spread and improving therapeutic efficacy remain hot topics in medical research worldwide. During tumor migration and invasion, EMT plays a critical role. Chen et al. found that Astragalus-Curcuma combination inhibits NETs expression by suppressing the Complement C5a/C5aR pathway, gradually ameliorating the hypercoagulable state of the body, thereby inhibiting tumor growth (Chen et al., 2003). Formononetin extracted from Astragalus root plays a significant role in inhibiting cancer cell proliferation, and metastasis by targeting key signaling pathways at interconnected junctions. It regulates cell cycle arrest and mediator protein-induced apoptosis, and promotes the upregulation of key regulatory factors (e.g., p-AKT and p53), while effectively inhibiting signaling pathways such as NF-κB. In addition, it can inhibit the expression of ERK1/2 pathway, which can further inhibit the signaling of lamin A/C, and ultimately regulate the tumor microenvironment (TME) to further inhibit tumor proliferation (Liu et al., 2025). Current studies have shown that formononetin can effectively inhibit the transduction of JAK/STAT signaling pathway and induce the inactivation of PKB/AKT pathway and MAPK pathway, which in turn can effectively inhibit the migration and invasion of cancer cells (Aliya et al., 2023). Jiang and colleagues et al. found that AS-IV inhibited the proliferative activity and invasiveness of breast cancer cells. Further studies revealed that AS-IV inhibited the activation of two key signaling molecules, ERK1/2 and JNK, members of the MAPK family, and downregulated the matrix metalloproteinases MMP-2 and MMP-9, which ultimately inhibited the invasive function of tumor cells. Furthermore, AS-IV mediates the downregulation of guanine nucleotide exchange factor Vav3 and inhibits activated Rac1 expression, ultimately suppressing tumor cell proliferation (Jiang et al., 2017).

*Critical Evaluation:* While these studies collectively suggest inhibitory effects of Astragalus metabolites on tumor proliferation and migration, most experiments were conducted exclusively *in vitro* using a limited range of tumor cell lines, often without inclusion of non-tumorigenic controls. The majority lacked dose–response analyses within clinically achievable concentration ranges, and *in vivo* validation remains scarce. Furthermore, mechanistic conclusions often rely on correlative signaling pathway changes without direct causality testing (e.g., gene knockdown or rescue experiments). These limitations reduce the strength of translational claims and highlight the need for standardized, multi-model validation approaches.

### 3.2 Targeting metabolic pathways to regulate tumor cell apoptosis

In tumor cells, the regulatory mechanisms of apoptosis are disrupted, leading to abnormal proliferation and survival. Key apoptotic regulators include Bcl-2 family proteins (Bcl-2, Bax) and caspase family proteins (caspase-3). Li et al. found that APS-mediated macrophages accelerate 4T1 cell apoptosis primarily via the mitochondrial apoptotic pathway without causing cytotoxicity (Li W. et al., 2020b; Jia et al., 2019). However, APS-mediated macrophages significantly inhibit 4T1 cell growth by inducing cell cycle arrest (G2 phase) and apoptosis. Other studies demonstrate that astragaloside IV enhances the Bax/Bcl-2 ratio, triggering endogenous apoptosis in various cancers, including breast, lung, colorectal (Zheng et al., 2019; Zhao Y. et al., 2019a; Cui et al., 2020). In liver cancer, breast cancer and bladder cancer cells, APS can significantly reduce mitochondrial membrane potential, increase the Bax/Bcl-2 ratio and cleave PARP. Simultaneously inhibit PI3K-p110β and p-AKT, promote FoxO1 translocation, thereby amplifying cisplatin-induced DNA damage, and increase the apoptosis rate from 12% to more than 40% (Chen et al., 2025). In cisplatin-refractory bladder cancer xenograft mice, APS combined with chemotherapy could reduce tumor volume by 55%, and no obvious systemic toxicity was observed. In the osteosarcoma model, APS inhibits JNK/c-Jun by upregulating miR-133a, significantly increasing the proportion of early and late apoptotic cells and inhibiting the metastasis trend (Chu et al., 2018). Astragalus saponin IV also exhibited broad-spectrum pro-apoptotic activity. In cells such AS colorectal cancer and non-small cell lung cancer, AS-IV can dose-dependently enhance the Bax/Bcl-2 ratio and activate caspase-3, and simultaneously downregulate Mcl-1 and NF-κB by blocking the IL-6/JAK2/STAT3 axis, thereby inhibiting anti-apoptotic signals. More importantly, AS-IV can upregulate Fas/FasL and DR5, enhance the sensitivity of tumor cells to TRAIL and radiotherapy, thereby overcoming the inherent tolerance of the exogenous death receptor pathway (Ran et al., 2025). In the breast cancer stem cell model, AS-IV also increased the paclitaxel-induced apoptosis rate by more than twice by inhibiting the Akt/GSK-3β/β-catenin signaling, significantly reducing the tumor burden of the stem cell subpopulation (Yang et al., 2024; Sun et al., 2023).

*Critical Evaluation:* Although apoptosis-related findings are promising, most studies rely on single cancer cell lines and short-term assays, making it difficult to assess long-term effects or off-target cytotoxicity. Concentrations of active compounds are often much higher than those achievable in human plasma, raising concerns about clinical relevance. *In vivo* evidence is limited, with few studies incorporating pharmacokinetic or biodistribution assessments. Moreover, mechanistic claims are often based on pathway inhibition or activation without definitive causal confirmation (e.g., knockdown/overexpression experiments or use of specific inhibitors). Future work should focus on multi-cell line validation, integration of physiological dosing, and comprehensive *in vivo* mechanistic testing.

### 3.3 Arrest of tumor cell cycle

Cell cycle arrest helps maintain genomic stability. Mutations in cell cycle regulatory genes play a significant role in tumorigenesis. Under normal conditions, DNA damage triggers cell cycle arrest at checkpoints, providing time for repair to minimize mutations and prevent tumor formation. Yang et al. studied Astragalus injection’s effects on proliferation, apoptosis, and cell cycle arrest in human nasopharyngeal carcinoma CNE-1 cells (Yang et al., 2021). CNE-1 cells were divided into Astragalus injection groups (50 g/L, 100 g/L, 200 g/L, 400 g/L, 800 g/L) and a control group. CCK-8 assays revealed dose- and time-dependent inhibition of proliferation. Flow cytometry showed cell cycle arrest at G0/G1 phase and increased apoptosis after 72 h, correlated with downregulated caspase-3 protein expression. Zhou et al. demonstrated that Astragalus injection inhibits CNE-2 cell proliferation via G0/G1 phase arrest and apoptosis induction, as confirmed by ATP-bioluminescence assays and flow cytometry (Zhou et al., 2017). Duan et al. found that Astragalus injection suppresses MCF-7 breast cancer cell growth by arresting the cell cycle at G1 phase (Duan et al., 2022). Deng and colleagues reported that miR-149 can inhibit the expression of EphB3 and downregulate the expression of PI3K/AKT signaling pathway, and then inhibit the function of STAT3 pathway, and ultimately induce G0/G1 stagnation in colorectal cancer cells, and inhibit the proliferation and invasion of colorectal cancer cells (Deng et al., 2021).

*Critical Evaluation:* Although related studies suggest that Astragalus injection and its active metabolites can induce tumor cell cycle arrest and promote apoptosis, most experiments are limited to *in vitro* cell models with small sample sizes and lack validation across diverse tumor types. Additionally, there is considerable variation in dosage ranges and exposure times, with a lack of *in vivo* or clinically relevant dosing validation, which limits the generalizability of the findings. Future studies should incorporate *in vivo* models and mechanistic investigations to improve reliability.

### 3.4 Targeting metabolic pathways to regulate tumor cell autophagy

Autophagy, a lysosome-mediated self-digestion process, recycles damaged organelles and long-lived proteins, playing dual roles in tumorigenesis. Early-stage autophagy suppresses tumors, while late-stage autophagy promotes tumor growth and metastasis by sustaining energy supply under stress (e.g., nutrient deprivation, TME) (Chen et al., 2023). Wang et al. found that astragaloside IV inhibits autophagy by modulating P62 and LC3 expression, suppresses MMP-2/MMP-9, enhances A549 cell adhesion, and reduces metastasis (Wang et al., 2018). Wang et al. showed that APS inhibits gastric cancer BGC823 cell proliferation, migration, and invasion, while increasing Beclin-1, Bax, and LC3-II/LC3-I ratios and decreasing p62 and Bcl-2, suggesting autophagy induction (Wang et al., 2017).

*Critical Evaluation:* Current literature shows some controversy regarding the role of Astragalus metabolites in regulating autophagy. Some studies report autophagy promotion, while others indicate inhibition, suggesting stage-specific and cell-type specific effects. Most studies lack *in vivo* validation and employ incomplete autophagy detection markers. There is a need to establish standardized and multi-dimensional evaluation systems.

### 3.5 Targeting metabolic reprogramming in the tumor microenvironment

The tumor microenvironment (TME), comprising diverse cells and biomolecules, is closely linked to tumor initiation, progression, and metastasis. Traditional therapies focus on tumor cells, but emerging strategies target tumor-host interactions. APS enhances immunity, modulates TME metabolites (cells, cytokines, extracellular matrix), suppresses inflammation, induces apoptosis, and reverses chemoresistance (Li Q. et al., 2024a). Bamodu et al. demonstrated that APS promotes dendritic cell maturation and M1 macrophage polarization in lung cancer TME, downregulating IL-10 (Bamodu et al., 2019). Lee et al. found APS inhibits VEGF and upregulates PD-1/PD-L1, aiding tumor immune escape (Lee et al., 2020). Elham et al. revealed APS boosts anti-tumor immunity in HeLa-PBMC co-cultures by inhibiting IL-10 and TGF-β (Elham et al., 2021).

*Critical Evaluation:* Research is generally limited to *in vitro* co-culture or isolated immune cell models, with a lack of systematic animal experiments and clinical data support. Immune regulatory mechanisms involve complex multi-layered signaling pathways, and most existing studies provide correlative observations without in-depth causal verification. Moreover, studies on dosage, safety, and long-term effects are insufficient.

### 3.6 Inhibition of tumor angiogenesis

VEGF-driven angiogenesis is critical for tumor vascularization. HUANG et al. showed APS normalizes tumor vasculature by blocking VEGF signaling, enhancing anti-cancer therapy efficacy (Huang et al., 2012). Zhao et al. found APS suppresses Lewis lung cancer growth/metastasis by downregulating VEGF and EGFR (Zhao L. et al., 2019b). Zhang et al. reported Astragalus flavonoids reduce VEGF and VEGFR-2 in tumor-bearing mice (Zhang Y. M. et al., 2024b). Law et al. demonstrated that Astragalus saponins inhibit mTOR signaling, downregulate VEGF, and suppress angiogenesis/metastasis (Law et al., 2012). Auyeung et al. revealed APS inhibits LoVo cell lymphangiogenesis via VEGF-C suppression (Auyeung et al., 2014). Astragaloside IV downregulates COX-2/PGE2/VEGF in SGC7901 and A549/U251 cells, curbing tumor growth (Chen et al., 2021).

*Critical Evaluation:* Although numerous *in vitro* and small animal model studies support the inhibitory effects of Astragalus metabolites on tumor angiogenesis, issues remain regarding inconsistent dosage standards, large variability among models, and lack of long-term efficacy and safety assessments. Molecular mechanism research mostly stays at the pathway level without verification of key targets.

### 3.7 Regulating metabolic pathways to enhance immune cell function

**FIGURE 2:**
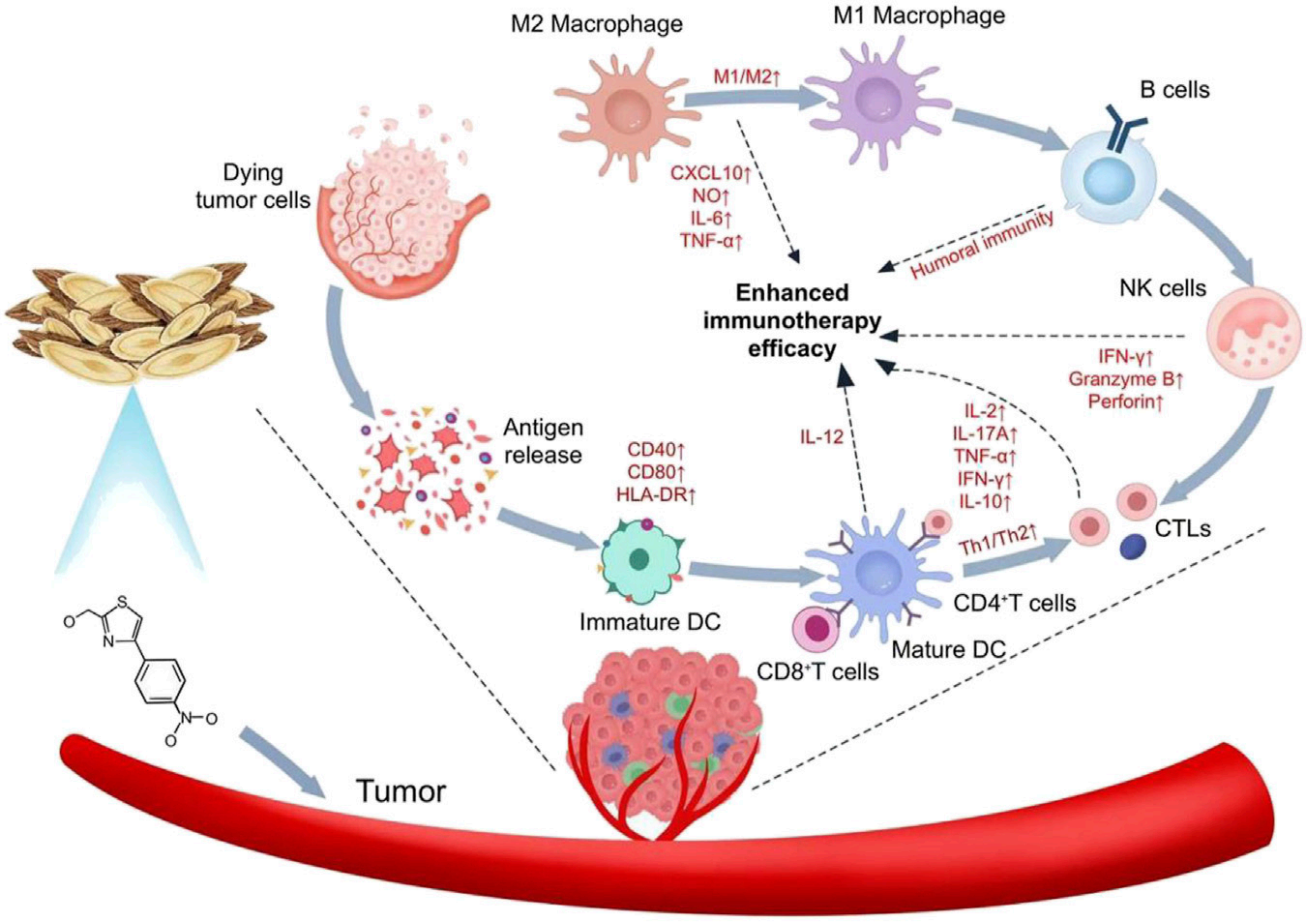
Mechanism of action of Astragalus in enhancing anti-tumor immunotherapy.

Some herbal preparations are widely used in the treatment of cancer due to their energizing and pro-apoptotic and cytotoxic effect, mainly by enhancing immunity (Chen Z. et al., 2024b). Tumor cells often rely on aerobic glycolysis and oxidative phosphorylation for energy acquisition. Given their rapid growth and high energy requirements, they experience increased glucose uptake as well as increased lactate production. Monocarboxylate transporter protein 1 and MCT4 are able to transport large amounts of lactate produced by tumor cells to the outside of the cell, which is critical for maintaining the acidic environment necessary for glycolysis in tumor cells (Hong et al., 2016). Zhang et al. showed that AS-IV was able to restore the expression of key molecules such as MCT1 and HIF-1α, and at the same time effectively inhibit the expression of the p53-mediated miRNA-34a/LDHA signaling pathway and TIGAR signaling pathway, which ultimately inhibited the development of gastric precancerous lesions (Zhang et al., 2018). Li et al. found astragaloside IV induces M1 macrophage polarization via STAT1 phosphorylation, activating anti-tumor immunity (Li M. et al., 2024b). Xu and colleagues found that AS-IV inhibited IL-13/IL-4 expression, which in turn inhibited induced M2 macrophage polarization and ultimately blocked M2 macrophage-driven A549 cell invasion and angiogenesis (Xu et al., 2018). Overall, Astragalus plays a major role in enhancing anti-tumor immunotherapy through different mechanisms of action (Figure 2).

*Critical Evaluation:* The immunometabolic regulatory effects of Astragalus are preliminarily established; however, most studies are limited to single cell lines or *in vitro* immune cell experiments, lacking comprehensive *in vivo* immune monitoring and clinical validation. The complexity of multiple intersecting metabolic pathways adds difficulty to mechanistic research, and future studies should integrate multi-omics approaches for systematic investigation.

## 4 Clinical applications of Astragalus

Astragalus is widely used in clinical practice as the main metabolites in various Chinese patent medicines and classical formulas, including decoctions, injections, granules, and other formulations. Its broad application across multiple clinical fields provides a strong scientific and rational basis for the use of Astragalus and its preparations (Yuan et al., 2022).

### 4.1 Classical formulas

Astragalus is a key metabolites in classical Chinese medicinal formulas, with 2,297 documented formulas containing Astragalus for treating 233 diseases (Liu et al., 2019). Classical Astragalus-based formulas, such as Huangqi Guizhi Wuwu Decoction (Astragalus and Cinnamon Twig Five-Substance Decoction), Buzhong Yiqi Decoction (Middle-Tonifying and Qi-Boosting Decoction), and Huangqi Jianzhong Decoction (Astragalus Center-Fortifying Decoction), are commonly used to alleviate tumor-related symptoms, enhance immune function, and reduce the incidence and severity of adverse reactions. According to TCM theory, tumorigenesis is associated with deficient “righteous qi” (vital energy). In Huangqi Guizhi Wuwu Decoction, Astragalus greatly supplements qi, strengthens immune function, and improves disease resistance. Chen studied 104 postoperative colorectal cancer patients, where the control group received FOLFOX4 chemotherapy, while the study group received additional Huangqi Guizhi Wuwu Decoction (Chen et al., 2014). Post-treatment serum levels of VEGF, TGF-β1, and MMP-9 in the study group were significantly lower than those in the control group, indicating that the decoction inhibits colorectal cancer cell secretion and metastasis, improves immune function, reduces cancer cell regeneration, enhances therapeutic efficacy, and promotes recovery. Yu compared the effects of mecobalamin (control group) with Huangqi Guizhi Wuwu Decoction combined with reverse acupuncture (observation group) on chemotherapy-induced peripheral neurotoxicity and immune function in malignant tumor patients (Yu et al., 2024). The observation group showed superior peripheral neurotoxicity grading and immune markers (CD4^+^, and CD8^+^ ratios). Chen et al. demonstrated that modified Huangqi Guizhi Wuwu Decoction significantly alleviates TP chemotherapy-induced peripheral neurotoxicity, reduces its incidence and severity, improves quality of life, and enhances chemotherapy tolerance (Chen et al., 2022b). Zeng et al. investigated the effects of Buzhong Yiqi Decoction combined with bevacizumab on tumor markers (CEA, CA125, CYFRA21-1) in advanced lung adenocarcinoma patients undergoing chemotherapy. The combination significantly reduced tumor marker levels and alleviated clinical symptoms (Zeng et al., 2024). Qiao et al. explored the role of Buzhong Yiqi Decoction combined with chemotherapy in improving serum tumor markers and nutritional status in advanced CRC patients (Qiao et al., 2025). A study by Xiao and colleagues et al. found that Huangqi Jianzhong Decoction in combination with chemotherapy was effective in relieving the side effects of treatment in patients with stage IIIb-IV gastric cancer and significantly promoted the efficacy of chemotherapy. Some efficacy was achieved in about 50% of the related gastric cancer patients. The combination reduced tumor markers (CEA, CA199, CA724), VEGF, and IL-6 levels, improved immune function and quality of life, and mitigated adverse drug reactions (Xiao et al., 2024). Hu et al. observed that Huangqi Jianzhong Decoction combined with apatinib elevated CD3^+^, CD4^+^, and CD4+/CD8+ ratios in advanced gastric cancer patients, enhancing cellular immune function (Hu et al., 2022) (Table 2).

**TABLE 2:** Classical Astragalus-based formulas: Composition, indications, and reporting in original studies.

Classical formula name	Main metabolites	Dosage information (if available)	Indications	Detailed composition and preparation reported in original studies?	References
Huangqi Guizhi Wuwu Decoction	Astragalus, Cinnamon Twig, White Peony, Angelica, Ligusticum	Dosages vary by study	Alleviation of tumor-related symptoms, immune enhancement	Partially reported	Chen et al. (2014), Yu et al. (2024), Chen et al. (2022b)
Buzhong Yiqi Decoction	Astragalus, Ginseng, Atractylodes, Angelica, Tangerine Peel, Licorice	Dosages vary by study	Adjuvant tumor therapy, immune function improvement	Partially reported	Zeng et al. (2024), Qiao et al. (2025)
Huangqi Jianzhong Decoction	Astragalus, Atractylodes, Licorice, Fresh Ginger, Jujube, Cinnamon Twig	Dosages vary by study	Relief of chemotherapy side effects in gastric cancer, quality of life improvement	Mostly reported	Xiao et al. (2024), Hu et al. (2022)

### 4.2 Astragalus preparations

Common modern Astragalus formulations include oral liquids, granules, Astragalus polysaccharide sterile powder for injection, and Astragalus injections. Recent advancements include Astragalus nanoparticles, which offer advantages such as small particle size, high surface area, enhanced reactivity, and strong adsorption capacity (Zhang et al., 2019). Compound Astragalus preparations include Shenqi Fuzheng Injection (Astragalus and Codonopsis), Zhenqi Fuzheng Capsules (Astragalus and Ligustrum), and Kang’ai Injection (Astragalus, Ginseng, and Matrine). Clinical trials confirm that these preparations alleviate cancer-related fatigue, improve gastrointestinal function, modulate inflammatory balance, reduce recurrence and metastasis rates, and enhance quality of life (Yu et al., 2018; Huang et al., 2020; Su et al., 2023).

### 4.3 Safety studies of Astragalus

Astragalus, used for millennia in TCM, is recorded as “non-toxic” in classical texts such as Mingyi Bielu (Supplementary Records of Famous Physicians), Bencaojing Jizhu (Commentary on the Classic of Materia Medica), and Bencaojing Jie (Interpretation of the Classic of Materia Medica) (Spallholz, 1994). Astragalus preparations and active metabolites exhibit low toxicity, with safety validated in clinical and preclinical studies. Recent pharmacological and toxicological research continues to evaluate its safety for broader clinical use. Szabo et al. conducted a 90-day toxicity study in Wistar rats administered Astragalus decoction (45–180 g/kg). No adverse effects were observed in general condition, hematology, urinalysis, organ weights, or histopathology, confirming long-term safety (Szabo, , 2014). Zhang et al. determined the LD50 of Astragalus injection in Kunming mice: intravenous LD50 was 90.39 g/kg (95% CI: 87.57–93.31 g/kg), and intraperitoneal LD50 was 108.11 g/kg (95% CI: 102.90–113.58 g/kg), with no significant acute or chronic toxicity (Zhang et al., 2020).

Astragalus has low acute and chronic toxicity and exhibits high safety even when used at high doses for short periods. However, some studies suggest that long-term high-dose use may be associated with organ toxicity, mutagenicity, and developmental toxicity. Caution is advised when using Astragalus in patients with impaired liver or kidney function and in pregnant women. This review summarizes the clinical application of Astragalus in pro-apoptotic and cytotoxic effects therapy (Table 3).

**TABLE 3:** Clinical trials related to Astragalus membranaceus in antitumor therapy.

NCT number	Study title	Study status	Conditions	Interventions	Phases
NCT01696565	PG2 Phase I/II Clinical Study Intravenously Administered in Patients With Advanced Malignancy	COMPLETED	Neoplasm Metastasis|Neoplasm Recurrence	DRUG: PG2	PHASE1|PHASE2
NCT06234072	Comparing Astragalus Plus Gemcitabine to Gemcitabine Alone as Neoadjuvant Treatment for Pancreatic Cancer Patients	NOT_YET_RECRUITING	Pancreatic Cancer	DRUG: Astragalus + Gemcitabine|DRUG: Gemcitabine alone	PHASE2
NCT06510530	Astragalus for Symptomatic Alleviation in High-grade Lymphoma	NOT_YET_RECRUITING	High-grade Lymphoma	DIETARY_SUPPLEMENT: Astragalus|DIETARY_SUPPLEMENT: Placebo	PHASE3
NCT01802021	Effect of Astragalus-based Formula: Qingshu-Yiqi-Tang on Modulating Immune Alterations in Lung Cancer Patients	UNKNOWN	Carcinoma, Non-Small-Cell Lung	DRUG: Astagalus-based Formula: Qingshu-Yiqi-Tang	PHASE2|PHASE3
NCT06557668	Clinical Outcomes of Treatment With Immunomodulator Plus Cancer Therapies for Patients With Colorectal Cancer	RECRUITING	Colorectal Cancer		NA
NCT01720550	PG2 Treatment for Improving Fatigue Among Advanced Cancer Patients Under Standard Palliative Care	COMPLETED	Cancer-related Fatigue	DRUG: Astragalus Polysaccharides 500 mg|DRUG: Astragalus Polysaccharides 250 mg	PHASE4
NCT03314805	PG2 Treatment Among Stage II/III Breast Cancer Patients Receiving Adjuvant Chemotherapy	COMPLETED	Cancer-related Fatigue|Neutropenia, Malignant	DRUG: Astragalus polysaccharides 500 mg|DRUG: Placebo|PROCEDURE: EC Chemotherapy	PHASE2
NCT01720563	A Phase II Trial of PG2 in Patients With Advanced Pharyngeal or Laryngeal Squamous Cell Carcinoma Under Concurrent Chemoradiotherapy	TERMINATED	Cancer-related Fatigue	DRUG: Astragalus polysaccharides 500 mg|DRUG: Placebo|PROCEDURE: Concurrent chemoradiotherapy with PUL (cisplatin/tegafur plus uracil (UFT)/leucovorin) every 2 weeks	PHASE2

## 5 Limitations and prospects

### 5.1 Clarification of findings and limitations

Current evidence indicates that Astragalus and its active metabolites exert notable cytotoxic and pro-apoptotic effects, primarily demonstrated through *in vitro* experiments and supported by some animal studies. Recent randomized controlled trials and meta-analyses further suggest that Astragalus may provide adjunctive benefits by alleviating chemotherapy-related adverse effects and improving quality of life in cancer patients. However, several critical limitations remain:

1. Most mechanistic studies are heavily reliant on *in vitro* models, with insufficient *in vivo* validation, which limits their translational relevance.
2. Considerable heterogeneity exists in experimental designs, including variations in cell lines, dosages, and treatment durations, complicating direct comparisons and meta-analytical synthesis.
3. Clinical studies frequently lack standardized outcome measures and consistent reporting, restricting the robustness of evidence synthesis.
4. The majority of clinical trials have been conducted in China, with limited independent replication in diverse international cohorts, raising concerns regarding potential publication bias and generalizability.

Therefore, well-designed *in vivo* studies, larger multicenter and multinational clinical trials, and standardized experimental and clinical protocols are urgently required to consolidate and extend the current understanding of Astragalus’ cytotoxic and pro-apoptotic activities.

### 5.2 Research status and safety considerations

As a traditional Chinese herbal medicine, the anti-tumor efficacy of Astragalus is undoubtedly significant. However, current research on its metabolites remains insufficient, largely due to the difficulty in separating and properly preserving its metabolites, as well as its low bioavailability. Additionally, given the complexity of traditional Chinese medicine’s syndrome differentiation and treatment, most studies are currently limited to basic research stages such as *in vitro* cell experiments and animal trials, while the quality of clinical research remains unsatisfactory (Li Z. et al., 2024c). Therefore, future studies urgently need to focus more effort on purification techniques and pharmacological property determination of Astragalus to deeply explore its medicinal value and enhance its clinical effectiveness and safety (Li et al., 2023). Attention should also be paid to the limitations of using quality markers to control the quality of Chinese medicine. These limitations mainly include their inability to reflect the holistic characteristics of interactions and synergistic effects among metabolites, as seen in formulas like Huangqi Guizhi Wuwu Decoction, Buzhong Yiqi Decoction, and Huangqi Jianzhong Decoction, which are important anti-tumor formulations. The effects of Chinese medicine, especially compound formulations, are complex. After compatibility, their functions become diverse, with varying dosages and decoction methods (Tang and Tian, 2024). The corresponding quality markers for different efficacy outcomes may also change, potentially creating a vicious cycle, as the dynamic balance between healthy and pathogenic factors often shifts (Xu et al., 2023).

Current clinical and animal evidence indicates that oral administration of Astragalus preparations at conventional doses is generally well tolerated, with no definitive reports of severe hepatotoxicity; notably, the LiverTox database records no significant elevations in serum transaminases or clinical drug-induced liver injury associated with Astragalus. Multiple *in vivo* studies have shown that total astragalus saponins or astragaloside IV can mitigate chemotherapy- or high-fat-diet–induced hepatic damage, suggesting a hepatoprotective potential. With respect to renal safety, randomized and prospective studies report modest improvements in estimated glomerular filtration rate (eGFR) in patients with chronic kidney disease (CKD) and no serious adverse events; however, Cochrane and other systematic reviews note that small sample sizes and short follow-up periods preclude definitive conclusions about long-term renoprotective effects (Li M.-Y. et al., 2025b). It should also be emphasized that commercial herbal supplements vary widely in quality and may contain heavy metals or other contaminants, which could impose additional hepatic and renal burdens with prolonged use. Consequently, Astragalus is regarded as a relatively safe medicine–food homology herb, but individuals with chronic hepatic or renal impairment, autoimmune disorders, or those requiring long-term high-dose therapy are advised to use it under medical supervision, with regular monitoring of ALT/AST and eGFR, and to select GMP-certified, standardized preparations to ensure feasibility and safety.

### 5.3 Methodological limitations and challenges

Despite the promising findings regarding the anti-tumor effects of Astragalus and its metabolites, several methodological limitations in the existing literature hinder definitive conclusions and clinical translation.

1. Inadequate Control Groups:
  Many studies lack rigorous control groups or appropriate placebo controls, limiting the ability to attribute observed effects specifically to Astragalus preparations rather than other confounding factors.
2. Insufficient Dose–Response Analyses:
  Few studies systematically explore the dose–response relationship, which is critical for determining effective and safe dosage ranges. The absence of such analyses complicates the optimization of treatment regimens and understanding of pharmacodynamics.
3. Lack of Mechanistic Investigations:
  While many reports demonstrate biological activities such as cytotoxicity and apoptosis induction, the underlying molecular mechanisms remain poorly characterized. This gap prevents a clear understanding of the multi-target effects and interaction networks involved.
4. Heterogeneity of Experimental Conditions:
  Significant variability exists in experimental models, extraction methods, compound standardization, and outcome measurements across studies. Such heterogeneity undermines reproducibility and comparability.

#### 5.3.1 Limited in vivo and clinical validation

Most evidence is derived from *in vitro* or animal models, with relatively few well-designed clinical trials. Translating findings from preclinical studies into clinical efficacy and safety remains a major challenge. Addressing these methodological issues in future research will strengthen the evidence base, facilitate regulatory approval, and enable more reliable incorporation of Astragalus-based therapies into clinical oncology.

### 5.4 Clinical evidence and limitation

In the past 5 years, multiple randomized controlled trials have shown that Astragalus membranaceus and its polysaccharides/injections are moving from the traditional “tonifying qi and nourishing the body” to the framework of evidence-based medicine. Research has found that the latest meta-analysis involving over 4,000 patients confirmed that the combination of Astragalus membranaceus alone or its compound preparations with standard anti-tumor regimens can significantly reduce cancer-related fatigue, bone marrow suppression and gastrointestinal toxicity, and simultaneously improve the objective response rate and quality of life score. This clinical benefit is not only reflected in common solid tumors such as breast cancer, non-small cell lung cancer (NSCLC), and colorectal cancer, but also extends to chronic complications such as chemoradiotherapy peripheral neuropathy and Long-COVID fatigue management. Astragalus Polysaccharide Injection PG2 is a single-active metabolites preparation. A multicenter, double-blind Phase II RCT (NCT03314805) demonstrated that intravenous infusion of PG2 500 mg on days 1, 3, and 8 of chemotherapy could reduce chemotherapy-related fatigue in patients with early breast cancer from a median of 7.4 points to 4.6 points, and significantly improve global health and sleep quality (Shen et al., 2024). There was no difference in safety events compared with placebo. In the context of immunotherapy, prospective cohort studies have reported that the combination of PG2 and PD-1 inhibitors can restore NSCLC patients with baseline NLR >5 to NLR<3 within 4 weeks, extend the progression-free survival from 5.5 months to 7.9 months, and there are no new grade III or IV adverse reactions. Compound injections such as Astragalus Injection and Aidi Injection can achieve the advantage of “multi-target synergy”. A study found that after adding Astragalus membranaceus to the treatment plan of NSCLC patients, the objective response rate of patients increased by 14%–18%, the bone marrow suppression of grade three and above decreased by approximately 30%, and the overall 1-year survival rate improved significantly (Chen J. et al., 2024c). A systematic review of 63 RCTS showed that the injection of traditional Chinese medicine containing Astragalus membranaceus combined with FOLFOX/XELOX in the treatment of advanced colorectal cancer could significantly increase the objective response rate (RR 1.34) and reduce grade IIIII - IV bone marrow suppression (RR 0.64) (Wang S. et al., 2021a). In the future, establishing Astragalus quality evaluation methods that link efficacy and safety, along with holographic pharmacodynamic substance-based quality evaluation systems, will better advance the development and utilization of Astragalus, effectively promote its clinical application in anti-tumor therapy, and actively improve the quality of life for cancer patients.

*Critical Evaluation:* Although recent clinical trials and meta-analyses provide encouraging evidence for the safety and efficacy of Astragalus preparations as adjuncts in cancer therapy, limitations remain. Many clinical studies suffer from heterogeneity in preparation standardization, dosing regimens, and patient populations, which complicates direct comparison and generalization. The majority of RCTs are conducted within China, and few are independently replicated in diverse global cohorts, raising concerns about potential publication bias and regional variability. Furthermore, mechanistic understanding of Astragalus’ multi-target effects remains incomplete, and the lack of robust pharmacokinetic and pharmacodynamic profiling impedes optimization of dosing and safety monitoring, especially in vulnerable populations. Quality control remains a major challenge due to the complex and dynamic nature of herbal metabolites and formula interactions. Future research must focus on rigorous standardization of herbal products, larger multicenter and multinational trials with longer follow-up, and integrated mechanistic studies to substantiate and optimize clinical applications. This will ensure that Astragalus can be more reliably incorporated into evidence-based oncology practice with maximized therapeutic benefit and minimized risks.

### 5.5 Future research directions

Based on the identified gaps and challenges in current studies, future research should prioritize the following aspects:

1. Establishment of standardized research protocols
  To ensure comparability and reproducibility, there is an urgent need to develop unified extraction methods, metabolites standards, and dosage specifications, especially to strictly follow standardized operating procedures in preclinical and clinical trials.
2. Integration of *in vitro* and *in vivo* models
  While most current studies focus on *in vitro* cell experiments, future efforts should strengthen validation in animal models and clinical samples to form a more comprehensive pharmacodynamic and safety evaluation pipeline.
3. Use of clinically relevant dosages and cell types
  Research should better reflect clinical realities by using drug concentrations within physiological ranges and tumor-relevant cell lines or tissues to enhance the translational value and practical guidance of the findings.
4. In-depth mechanistic investigations
  The multi-target and multi-metabolites mechanisms of Astragalus remain incompletely elucidated. Future studies should employ omics technologies and systems biology approaches to uncover the interaction networks of Astragalus and its metabolites within the tumor microenvironment.
5. Large-scale, multicenter clinical trials
  To address limitations such as small sample sizes and regional concentration in existing clinical studies, there is a need to conduct large, multicenter, and international randomized controlled trials to validate the clinical efficacy and safety of Astragalus preparations.

Addressing these key issues will lay a solid foundation for the standardized and evidence-based clinical application of Astragalus in anti-tumor therapy.

## 6 Conclusion

The active metabolites of Astragalus, including polysaccharides, flavonoids, and saponins, exhibit multi-target effects with low toxicity, contributing to the inhibition of tumor proliferation and migration, induction of apoptosis and autophagy, cell cycle arrest, modulation of the tumor microenvironment, and enhancement of host immunity. However, clinical evidence supporting the use of Astragalus in oncology remains limited due to heterogeneous trial quality, insufficient standardization of preparations, a lack of pharmacokinetic and toxicological data, and unclear mechanisms of interaction with modern anticancer therapies. Future research should prioritize rigorously designed multicenter phase III randomized controlled trials to quantify clinical benefits, combined with mechanistic analyses using single-cell and spatial multi-omics as well as network pharmacology to identify precise targets and interaction networks. Development of nano-delivery systems, sustained-release formulations, and tumor-targeted preparations could enhance bioavailability and tumor accumulation. Systematic pharmacokinetic and drug-interaction studies are also warranted to optimize safety. Furthermore, stratification of patients based on immunophenotypes and biomarkers, along with exploration of optimal combinations with immune checkpoint inhibitors, PARP inhibitors, and anti-angiogenic agents, may maximize therapeutic benefit. Through precise, multi-disciplinary, and evidence-based research, Astragalus could evolve from a traditional adjuvant to a comprehensive adjunctive regimen, laying a solid foundation for improving the quality of life and long-term outcomes of cancer patients.

## Appendix

**TABLE A1:** Verified plant species names and taxonomic details

Scientific name	Authority	Family	Medicinal material name
Astragalus membranaceus Fisch	Fisch	Fabaceae (Leguminosae)	Astragalus Root (Huangqi Root)
Astragalus mongholicus Bunge	Bunge	Fabaceae	Mongolian Astragalus Root
Astragalus complanatus Bunge	Bunge	Fabaceae	Flattened Astragalus
Astragalus membranaceus var. mongholicus	(Bunge) P.K.Hsiao	Fabaceae	Mongolian Astragalus (variety)
